# Pre-hospital predictors of an adverse outcome among patients with dyspnoea as the main symptom assessed by pre-hospital emergency nurses - a retrospective observational study

**DOI:** 10.1186/s12873-020-00384-1

**Published:** 2020-11-10

**Authors:** Wivica Kauppi, Johan Herlitz, Thomas Karlsson, Carl Magnusson, Lina Palmér, Christer Axelsson

**Affiliations:** 1 PreHospen- Centre for Pre-hospital Research, Faculty of Caring, Work Life and Social Welfare, University of Borås, SE-501 90 Borås, Sweden; 2 Faculty of Caring Science, Work Life and Social Welfare, University of Borås, Borås, Sweden; 3 Biostatistics, School of Public Health and Community Medicine, Institute of Medicine, Sahlgrenska Academy, University of Gothenburg, Gothenburg, Sweden; 4 Department of Molecular and Clinical Medicine, Sahlgrenska Academy, University of Gothenburg, Gothenburg, Sweden

**Keywords:** Dyspnoea, Epidemiology, Adverse outcome, Time-sensitive diagnosis, Ambulance, Emergency medical service, Pre-hospital emergency nurse

## Abstract

**Background:**

Dyspnoea is one of the most common reasons for patients contacting emergency medical services (EMS). Pre-hospital Emergency Nurses (PENs) are independently responsible for advanced care and to meet these patients individual needs. Patients with dyspnoea constitute a complex group, with multiple different final diagnoses and with a high risk of death. This study aimed to describe on-scene factors associated with an increased risk of a time-sensitive final diagnosis and the risk of death.

**Methods:**

A retrospective observational study including patients aged ≥16 years, presenting mainly with dyspnoea was conducted. Patients were identified thorough an EMS database, and were assessed by PENs in the south-western part of Sweden during January to December 2017. Of 7260 missions (9% of all primary missions), 6354 were included. Among those, 4587 patients were randomly selected in conjunction with adjusting for unique patients with single occasions. Data were manually collected through both EMS- and hospital records and final diagnoses were determined through the final diagnoses verified in hospital records. Analysis was performed using multiple logistic regression and multiple imputations.

**Results:**

Among all unique patients with dyspnoea as the main symptom, 13% had a time-sensitive final diagnosis. The three most frequent final time-sensitive diagnoses were cardiac diseases (4.1% of all diagnoses), infectious/inflammatory diseases (2.6%), and vascular diseases (2.4%). A history of hypertension, renal disease, symptoms of pain, abnormal respiratory rate, impaired consciousness, a pathologic ECG and a short delay until call for EMS were associated with an increased risk of a time-sensitive final diagnosis. Among patients with time-sensitive diagnoses, approximately 27% died within 30 days. Increasing age, a history of renal disease, cancer, low systolic blood pressures, impaired consciousness and abnormal body temperature were associated with an increased risk of death.

**Conclusions:**

Among patients with dyspnoea as the main symptom**,** age, previous medical history, deviating vital signs, ECG pattern, symptoms of pain, and a short delay until call for EMS are important factors to consider in the prehospital assessment of the combined risk of either having a time-sensitive diagnosis or death.

**Supplementary Information:**

**Supplementary information** accompanies this paper at 10.1186/s12873-020-00384-1.

## Background

Dyspnoea, also known as shortness of breath or breathlessness, is one of the most common reasons patients contact EMS. Patients with dyspnoea constitute a heterogeneous group since comorbidity is common. Dyspnoea is associated with a high risk of death and is caused by several medical conditions that include physiological, pathological, psychological conditions, or social reasons [1, 2]. In Sweden, PENs have an important role in the assessment and triage, as well as in the care of patients with dyspnoea on the scene. This assessment challenges the PENs ability to assess the patient properly [3], which requires a comprehensive understanding of the pathophysiology, as well as the ability to meet the patients existential needs since anxiety is common in connection with dyspnoea [4]. Few epidemiological studies have described patients with dyspnoea in pre-hospital settings [5–7]. A recent study by Kauppi et al. [8] described the epidemiology and outcomes of patients with dyspnoea as the main symptom from a larger perspective. In the current study among patients whose main symptom was dyspnoea, we aimed to describe a) time-sensitive conditions in further detail and b) the factors that already on scene were associated with the risk of either having a time-sensitive final diagnosis or death within 30 days when receiving care from PENs. The two primary endpoints were thus: 1. A time-sensitive condition according to the final diagnosis, and 2. Death within 30 days.

## Methods

The results in this study are based on further analysis from the same study population as recently described by Kauppi et al. [8].

### Study design

This is an exploratory observational study with a retrospective manual assessment of EMS and hospital records by one single person. The study included all patients who dialled 112 in Sweden between January and December 2017 with dyspnoea as the main symptom. The call was followed by an ambulance being dispatched to the scene, and the provision of assessment and care by PENs.

### Study settings

**Fig. 1:**
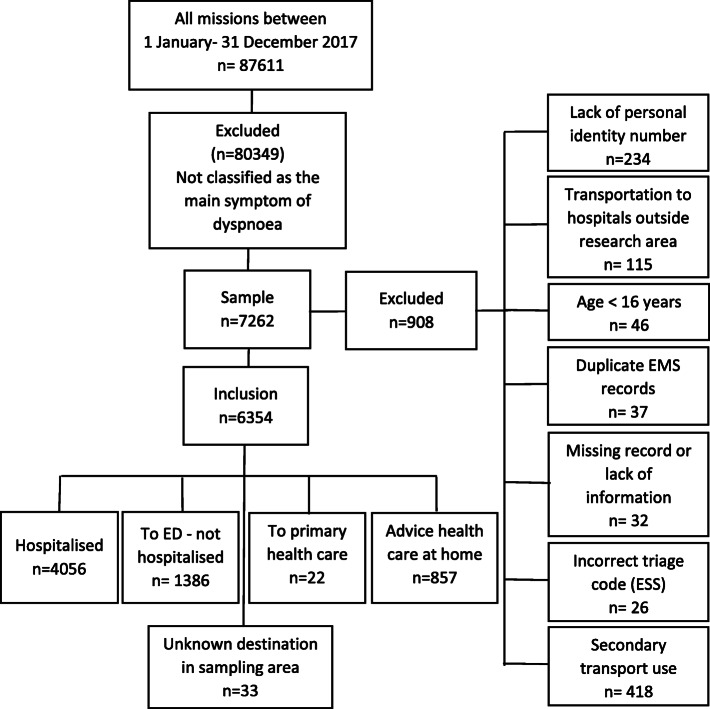
Flow chart of all studied patients, assessed as the main symptom of dyspnoea

The study included two EMS organisations in the south-western part of Sweden with a catchment population of 962,000 inhabitants and a catchment area of approximately 7400 km^2^ including both urban, suburban and rural areas. In all, the EMS organisations had 123,614 missions with a priority level of 1 to 3. Among them, 87,611 missions where involved in an initial patient assessment that was defined as a primary mission (Fig. 1). In Sweden, all ambulances are since 2005 at a minimum staffed with two health care providers, of which one is a registered nurse [9]. A major part of the nurses have fulfilled a three-year nursing course as well as a one-year master’s course that are focusing on pre-hospital emergency care and are given the professional title ‘PEN’. The PENs are responsible for care including assessment and treatment according to local and national guidelines.

### Patients

Patients were included by missions through an EMS-record database and a hospital record system. The EMS recorded database includes the triage classification system (RETTS-A) and the hospital record system contains the International Classification of Diseases (ICD-10) codes, which includes 22 chapters (I–XXII). Electrocardiogram (ECG) interpretations were primarily collected from pre-hospital records, alternatively from Emergency Department (ED) notes, and if not available, interpreted by the first author (WK). ECG abnormalities that were looked for included atrial flutter, atrial fibrillation, ST-segment and T-wave deviation, left bundle branch block, ventricular tachycardia, ectopic atrial rhythm or tachycardia, and AV-block. Of all vital signs (VS), the first (on arrival at the patient’s side) and last (before admission to ED) assessments were used.

**Fig. 2:**
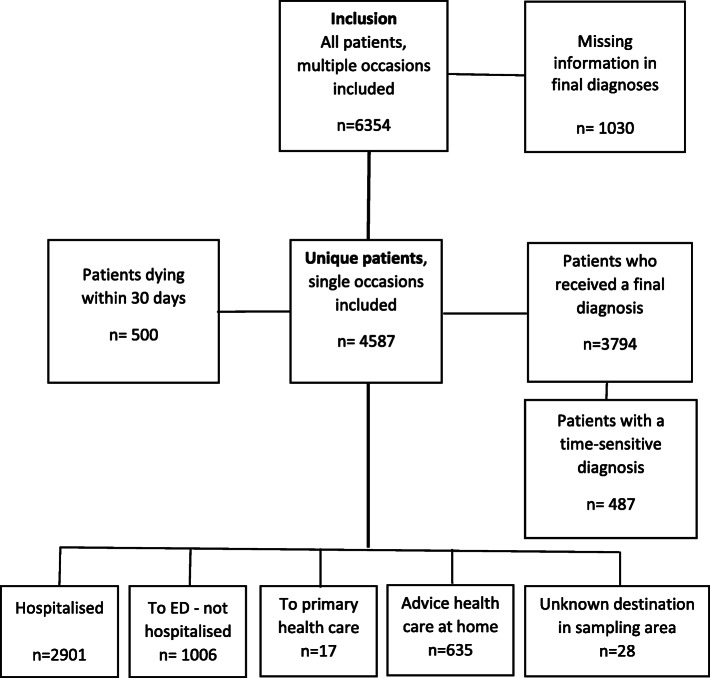
Flow chart of the included unique patients, single occasions randomly selected, assessed as the main symptom of dyspnoea

Inclusion criteria were a primary mission where the dispatch centre had been contacted and was given a priority level of 1–3, and where the mission was classified on scene as dyspnoea as the main symptom, which consists of the Emergency Symptoms and Signs (ESS) code number 04. Exclusion criteria included age < 16 years, no personal identity number, an incorrect ESS triage code, transportation to a hospital outside the catchment areas, limited information, EMS records that appeared more than once and the use of a secondary transport. A total of 7260 patients were identified (9% of all missions) of which 908 patients were excluded. Thus, altogether 6354 patients took part in the final analyses, some presenting on multiple occasions (Fig. 1). Among these 6354 patients were 4587 randomly selected adjusting for unique patients with single occasions (Fig. 2).

### Triage system

The triage classification system Rapid Emergency Triage and Treatment System for adults (RETTS- A) is used by the PENs to assess and prioritise patients degree of pre-hospital care required [10]. This system is based on VS and ESS, which both (independently of each other) leads to a recommendation of the priority level. The highest level of either the ESS or VS determines the final triage level [see Additional file 1: Red and orange VS and ESS code 04, dyspnoea]. The priority level is expressed in five different colours (red, orange, yellow, green, and blue), which refers to time from the assessment until time when patients require to be assessed by a physician. Red and orange level indicate the highest priority (life-threatening vs. potentially life-threatening), whereas the remaining colours represent situations when there is no medical risk for patients if the waiting time is prolonged before physician assessment. In the pre-hospital triage, the level blue is not used.

### Time-sensitive diagnosis

We defined a time-sensitive diagnosis as a diagnosis when urgent care and treatment are vital to limit organ damage and to avoid severe complications and early death (e.g. pulmonary oedema, acute myocardial infarction and sepsis) [3, 11]. This definition has been previously described [3], but some additional diagnoses (e.g. acute respiratory failure and acidosis) that fulfilled the criteria for the above definition were added in the analyses of a recent cohort study by Kauppi et al. [8] and have therefore been included.

### Statistical analysis

Multiple logistic regression was used for calculation of age-adjusted *p*-values and odds ratios with corresponding confidence intervals regarding time-sensitive diagnosis and 30-day mortality, respectively. Available data was used for these calculations.

To identify factors independently associated with the two outcomes, all variables with an age-adjusted *p* < 0.20 in Tables 2 and 4, respectively, were tested for inclusion in a final multiple logistic regression model. No significant collinearity was found between the variables by association measures, as well as by inspection of the variance inflation factor, condition index, and eigenvector proportions in a multiple linear regression model including all the candidate variables. Due to the amount of missing data for several of the variables, multiple imputations were applied. For these analyses, missing data were assumed to be missing at random (MAR) and 50 imputed datasets were generated with the Markov Chain Monte Carlo (MCMC) method using the expectation-maximization (EM) algorithm. Rubin’s rules were used for pooling the results from the imputed datasets. To identify variables independently associated with outcome, we started with a model including all the variables as identified above. Multiple logistic regression was performed in each of the 50 imputed datasets, and the variable with the highest *p*-value in the pooled result was excluded from the model. A new regression analysis was then performed in each imputed dataset and of the remaining variables, the one with the highest p-value in the pooled result was excluded. This procedure was repeated until all remaining variables yielded a p-value below 0.01 in the pooled result. This was performed separately for the time-sensitive diagnosis and 30-day mortality outcomes. Two-sided tests were used and *p*-values below 0.01 were considered statistically significant. All analyses were performed using SAS for Windows version 9.4.

## Results

In all, 6354 EMS missions were included (Fig. 1). Among them, there were 4587 unique patients with dyspnoea as the main symptom, of which 3794 had a final diagnosis. Time-sensitive final diagnoses were reported in 487 cases, which was approximately 13% of all unique patients with a final diagnosis (Fig. 2), and among them approximately 27% died within 30 days.

### Occurrence of time-sensitive diagnosis among all missions with a final diagnosis

The most frequent final time-sensitive diagnoses among all missions were cardiac diseases (4.1% of all diagnoses) followed in order of frequency by infectious/inflammatory diseases (2.6%), vascular diseases (2.4%), respiratory diseases (1.5%), and neurological diseases (0.3%).

The cardiac and infectious time-sensitive diagnoses were more common in males and the elderly whereas the vascular and the respiratory time-sensitive diagnoses were most frequent in females and younger patients. Of all missions with a final diagnosis (*n* = 5324 missions), 11.5% had a time-sensitive diagnosis (Table 1).

**Table 1:** Occurrence of time-sensitive diagnosis among all missions with a final diagnosis

	All patients (*n* = 5324)^a^	Women (*n* = 2905)	Men (*n* = 2419)	Age ≤ 77^b^ (*n* = 2514)	Age > 77 (*n* = 2810)
Cardiac	219 (4.1)	102 (3.5)	117 (4.8)	85(3.4)	134 (4.8)
Myocardial infarction	127 (2.4)	57 (2.0)	70 (2.9)	44 (1.8)	83 (3.0)
Cardiac arrest	1 (< 0.1)	1 (< 0.1)	0 (0.0)	0 (0.0)	1 (< 0.1)
Pulmonary oedema	83 (1.6)	43 (1.5)	40 (1.7)	34 (1.4)	49 (1.7)
Unstable angina	8 (0.2)	1 (< 0.1)	7 (0.3)	7 (0.3)	1 (< 0.1)
Vascular	130 (2.4)	83 (2.9)	47 (1.9)	72 (2.9)	58 (2.1)
Pulmonary embolism	123 (2.3)	81 (2.8)	42 (1.7)	70 (2.8)	53 (1.9)
Vessel embolism	4 (0.1)	1 (< 0.1)	3 (0.1)	2 (0.1)	2 (0.1)
Aortic dissection	1 (< 0.1)	1 (< 0.1)	0 (0.0)	0 (0.0)	1 (< 0.1)
Aortic rupture	2 (< 0.1)	0 (0.0)	2 (0.1)	0 (0.0)	2 (0.1)
Infection and inflammation	137 (2.6)	64 (2.2)	73 (3.0)	56 (2.2)	81 (2.9)
Sepsis	111 (2.1)	50 (1.7)	61 (2.5)	43 (1.7)	68 (2.4)
Epiglottitis	1 (< 0.1)	0 (0.0)	1 (< 0.1)	1 (< 0.1)	0 (0.0)
SIRS^c^	17 (0.3)	11 (0.4)	6 (0.2)	8 (0.3)	9 (0.3)
Other infection^d^	8 (0.2)	3 (0.1)	5 (0.2)	4 (0.2)	4 (0.1)
Respiratory					
Acute respiratory insufficiency	78 (1.5)	47 (1.6)	31 (1.3)	47 (1.9)	31 (1.1)
Neurological	14 (0.3)	7 (0.2)	7 (0.3)	3 (0.1)	11 (0.4)
Status epilepsia	1 (< 0.1)	0 (0.0)	1 (< 0.1)	1 (< 0.1)	0 (0.0)
Stroke	11 (0.2)	5 (0.2)	6 (0.2)	2 (0.1)	9 (0.3)
TIA	2 (< 0.1)	2 (0.1)	0 (0.0)	0 (0.0)	2 (0.1)
Other^e^	20 (0.4)	7 (0.2)	13 (0.5)	18 (0.7)	2 (0.1)

Number (percent);

^a^ Patients with multiple occasions included; number missing diagnosis *n* = 1030

^b^ Median age in years

^c^ Systemic inflammatory response syndrome (SIRS) of non-infectious origin with acute dysfunction

^d^ Pneumonitis, pericarditis, perotonitis, hemorrhagic fever

^e^ Various reasons: acidosis, acute kidney failures, acute intoxications

### Age-adjusted relationships between time-sensitive diagnosis and sex, previous history, time intervals, and clinical observations on arrival of PENs, as well as age itself, among unique patients with a final diagnosis

Time-sensitive diagnoses among unique patients with a final diagnosis were reported in approximately 13% of all cases. Among them, the most common clinical deviations from normal were an abnormal respiratory rate (72.3%), a low oxygen saturation (59.5%), and a pathological ECG (52.6%). The following were significantly associated with an increased risk of a time-sensitive diagnosis: a history of hypertension, diabetes and renal disease, recent or ongoing syncope, abnormalities in any of the six VS, and a pathological ECG.

The following were significantly associated with a decreased risk: a history of dyspnoea, atrial fibrillation, and pulmonary disease, increasing interval from onset of symptoms until the call for EMS and time from the call for EMS to PENs arrival (Table 2).

**Table 2:** Age- adjusted relationships between time-sensitive diagnosis and sex, previous history, time intervals, and clinical observations on arrival of PENs, as well as age itself, among unique patients with a final diagnosis

		Time-sensitive diagnosis			
	All patients (*n* = 3794)^a^	Yes (*n* = 487)	No (*n* = 3307)	Age adjusted	
				p	OR (95% CI)
Age	79 (53,91)	80 (61,91)	79 (52,91)	0.02	
< =65	753 (19.8)	72 (14.8)	681 (20.6)		1
66–80	1327 (35.0)	183 (37.6)	1144 (34.6)		1.51 (1.13,2.02)
> 80	1714 (45.2)	232 (47.6)	1482 (44.8)		1.48 (1.12,1.96)
Male sex	1746 (46.0)	239 (49.1)	1507 (45.6)	0.11	1.17 (0.96,1.41)
Dyspnoea (319)*	2867 (82.5)	324 (76.2)	2543 (83.4)	< 0.0001	0.58 (0.46,0.75)
Ischemic heart disease (21)	1017 (27.0)	149 (30.7)	868 (26.4)	0.16	1.17 (0.94,1.44)
Heart failure (19)	1163 (30.8)	159 (32.7)	1004 (30.5)	0.78	1.03 (0.84,1.27)
Hypertension	1905 (50.2)	280 (57.5)	1625 (49.1)	0.007	1.32 (1.08,1.61)
Diabetes	796 (21.0)	130 (26.7)	666 (20.1)	0.002	1.41 (1.13,1.76)
Atrial fibrillation (1)	1168 (30.8)	116 (23.8)	1052 (31.8)	< 0.0001	0.59 (0.47,0.75)
Pulmonary disease (72)	1537 (41.3)	127 (26.5)	1410 (43.5)	< 0.0001	0.45 (0.36,0.56)
Renal disease (1)	465 (12.3)	79 (16.2)	386 (11.7)	0.01	1.40 (1.08,1.83)
System disease (4)	270 (7.1)	30 (6.2)	240 (7.3)	0.32	0.82 (0.55,1.22)
Cancer (8)	767 (20.3)	88 (18.1)	679 (20.6)	0.09	0.80 (0.63,1.03)
Psychiatric disorder (11)	615 (16.3)	62 (12.8)	553 (16.8)	0.06	0.76 (0.58,1.02)
Pain (178)	913 (25.2)	127 (28.4)	786 (24.8)	0.06	1.24 (0.99,1.55)
Syncope (18)	53 (1.4)	15 (3.1)	38 (1.2)	0.0005	2.95 (1.60,5.42)
Respiratory rate < 8 or > 25 (breaths/min)(81)	2020 (54.4)	345 (72.3)	1675 (51.8)	< 0.0001	2.38 (1.92,2.95)
Oxygen saturation < 90 (%) (74)	1446 (38.9)	282 (59.5)	1164 (35.9)	< 0.0001	2.56 (2.10,3.13)
Systolic blood pressure < 90 (mmHg)(133)	84 (2.3)	25 (5.4)	59 (1.8)	< 0.0001	3.03 (1.87,4.89)
Heart rate < 40 or > 120 (beats/min)(80)	488 (13.1)	116 (24.6)	372 (11.5)	< 0.0001	2.59 (2.04,3.28)
Body temperature < 35.0 or > 41.0 (°C)(129)	23 (0.6)	8 (1.8)	15 (0.5)	0.002	3.95 (1.66,9.42)
Degree of consciousness (RLS) > 2 (694)	67 (2.2)	31 (8.1)	36 (1.3)	< 0.0001	6.55 (3.99,10.74)
Pathological ECG (284)	1110 (31.6)	243 (52.6)	867 (28.4)	< 0.0001	2.76 (2.26,3.37)
Time from symptom onset to call (193)	58 (1340)	27 (0,223)	62 (1341)	< 0.0001	
< =12 h	1076 (29.9)	185 (41.1)	891 (28.3)		1
12–24 h	179 (5.0)	22 (4.9)	157 (5.0)		0.66 (0.41,1.06)
24–48 h	320 (8.9)	36 (8.0)	284 (9.0)		0.60 (0.41,0.88)
48–72 h	391 (10.9)	54 (12.0)	337 (10.7)		0.75 (0.54,1.04)
> 72 h	1635 (45.4)	153 (34.0)	1482 (47.0)		0.49 (0.39,0.61)
Time from call to PENs arrival (51)	18 (9,51)	15 (8,43)	18 (9.51)	< 0.0001	
0–6 min	90 (2.4)	18 (3.8)	72 (2.2)		1
7–12 min	929 (24.8)	155 (32.5)	774 (23.7)		0.79 (0.46,1.36)
13–24 min	1527 (40.8)	176 (36.9)	1351 (41.4)		0.51 (0.30,0.88)
> 24 min	1197 (32.0)	128 (26.8)	1069 (32.7)		0.47 (0.27,0.81)

Median (10th,90th percentile) or number (percent); * number missing

^a^ Unique patients, randomly selected occasions included

### Multivariable analysis of factors independently associated with a time-sensitive diagnosis using multiple imputations (3794 patients, 487 with a time-sensitive diagnosis)

The following were significantly independently associated with an increased risk of a time-sensitive diagnosis among unique patients: a history of hypertension and renal disease, symptoms of pain, an abnormal respiratory rate, a low oxygen saturation, an abnormal heart rate, a decreased level of consciousness and a pathologic ECG.

The following were associated with a decreased risk: a history of atrial fibrillation and pulmonary disease and an increasing delay from symptom onset until the call for EMS (Table 3).

**Table 3:** Multivariable analysis of factors independently associated with a time-sensitive diagnosis using multiple imputations (3794 patients, 487 with a time-sensitive diagnosis)

	Multiple imputations	
	OR^a^ (95%CI) ^a^	p
Hypertension	1.45 (1.17,1.79)	0.0006
Atrial fibrillation	0.55 (0.43,0.70)	< 0.0001
Pulmonary disease	0.42 (0.33,0.53)	< 0.0001
Renal disease	1.53 (1.15,2.05)	0.004
Pain	1.42 (1.12,1.81)	0.004
Respiratory rate < 8 or > 25 (breaths/min)	1.75 (1.37,2.22)	< 0.0001
Saturation < 90 (%)	2.02 (1.61,2.53)	< 0.0001
Heart rate < 40 or > 120 (rate/min)	2.08 (1.60,2.70)	< 0.0001
Degree of consciousness (RLS) > 2	4.83 (2.77,8.44)	< 0.0001
Pathological ECG	2.46 (1.99,3.03)	< 0.0001
Time from symptom to call		0.004
< =12 h	1	
12–24 h	0.69 (0.41,1.15)	
24–48 h	0.62 (0.41,0.93)	
48–72 h	0.86 (0.61,1.22)	
> 72 h	0.64 (0.50,0.82)	

^a^ odds ratio with corresponding 95% confidence interval

### Age-adjusted relationships between 30- day mortality and sex, previous history, time intervals, and clinical observations on the arrival of PENs among unique patients

Approximately 11% of all unique patients died within 30 days. Among them, the most common deviations from normal were an abnormal respiratory rate (67.9%), a low oxygen saturation (60.3%), and a pathological ECG (38.1%).

The following were associated with an increased risk of death: increasing age, male sex, a history of renal disease and cancer, deviation from normal in any of the six VS, and a pathologic ECG. Only a history of pulmonary disease was associated with a decreased risk (Table 4).

**Table 4:** Age- adjusted relationships between 30- day mortality and sex, previous history, time intervals, and clinical observations on arrival of PENs among unique patients

		Dead within 30 days			
	All patients (*n* = 4587)^a^	Yes (*n* = 500)	No (*n* = 4087)	Age adjusted	
				p	OR (95% CI)
Age	77 (43,91)	83 (68,93)	76 (39,90)	< 0.0001	
< =65	1197 (26.1)	38 (7.6)	1159 (28.4)		1
66–80	1505 (32.8)	170 (34.0)	1335 (32.7)		3.88 (2.71,5.57)
> 80	1885 (41.1)	292 (58.4)	1593 (39.0)		5.59 (3.95,7.90)
Male sex	2051 (44.7)	252 (50.4)	1799 (44.0)	0.002	1.36 (1.12,1.64)
Dyspnoea (418)*	3302 (79.2)	371 (83.4)	2931 (78.7)	0.96	0.99 (0.76,1.30)
Ischemic heart disease (21)	1140 (25.0)	131 (26.3)	1009 (24.8)	0.07	0.82 (0.66,1.02)
Heart failure (31)	1262 (27.7)	182 (36.7)	1080 (26.6)	0.16	1.16 (0.94,1.41)
Hypertension (8)	2114 (46.2)	256 (51.2)	1858 (45.6)	0.15	0.87 (0.71,1.05)
Diabetes (12)	878 (19.2)	106 (21.2)	772 (18.9)	0.76	1.04 (0.82,1.31)
Atrial fibrillation (13)	1277 (27.9)	175 (35.1)	1102 (27.0)	0.92	1.01 (0.82,1.24)
Pulmonary disease (89)	1789 (39.8)	169 (34.2)	1620 (40.5)	0.004	0.74 (0.61,0.91)
Renal disease (12)	499 (10.9)	83 (16.6)	416 (10.2)	0.006	1.44 (1.11,1.87)
System disease (15)	307 (6.7)	40 (8.0)	267 (6.6)	0.55	1.11 (0.78,1.58)
Cancer (17)	863 (18.9)	149 (29.9)	714 (17.5)	< 0.0001	1.67 (1.35,2.07)
Psychiatric disorder (30)	828 (18.2)	74 (14.8)	754 (18.6)	0.84	0.97 (0.75,1.27)
Pain (206)	1098 (25.1)	89 (19.6)	1009 (25.7)	0.05	0.78 (0.61,0.99)
Syncope (19)	63 (1.4)	9 (1.8)	54 (1.3)	0.20	1.62 (0.78,3.36)
Respiratory rate < 8 or > 25(breaths/min)(105)	2104 (46.9)	332 (67.9)	1772 (44.4)	< 0.0001	2.19 (1.78,2.68)
Oxygen saturation < 90 (%) (90)	1485 (33.0)	296 (60.3)	1189 (29.7)	< 0.0001	3.06 (2.52,3.73)
Systolic blood pressure < 90 (mmHg) (178)	90 (2.0)	37 (7.8)	53 (1.3)	< 0.0001	6.45 (4.12,10.10)
Heart rate < 40 or > 120 (beats/min) (95)	517 (11.5)	84 (17.1)	433 (10.8)	< 0.0001	1.80 (1.39,2.34)
Body temperature < 35.0 or > 41.0 (°C) (187)	24 (0.5)	10 (2.1)	14 (0.4)	< 0.0001	6.38 (2.70,15.04)
Degree of consciousness (RLS) > 2 (816)	70 (1.9)	27 (7.2)	43 (1.3)	< 0.0001	5.56 (3.35,9.23)
Pathological ECG (690)	1153 (29.6)	173 (38.1)	980 (28.5)	0.002	1.38 (1.13,1.70)
Time from symptom to call (332)	51 (0,339)	50 (0,318)	51 (0,340)	0.41	
< =12 h	1470 (34.5)	163 (34.8)	1307 (34.5)		1
12–24 h	210 (4.9)	20 (4.3)	190 (5.0)		0.79 (0.48,1.30)
24–48 h	357 (8.4)	37 (7.9)	320 (8.5)		0.86 (0.59,1.26)
48–72 h	420 (9.9)	50 (10.7)	370 (9.8)		0.94 (0.67,1.33)
> 72 h	1798 (42.3)	199 (42.4)	1599 (42.2)		0.89 (0.71,1.12)
Time from call to EMS arrival (58)	18 (9,50)	16 (8,44)	18 (9,51)	0.02	
0–6 min	100 (2.2)	16 (3.2)	84 (2.1)		1
7–12 min	1110 (24.5)	135 (27.2)	975 (24.2)		0.69 (0.39,1.23)
13–24 min	1853 (40.9)	198 (39.8)	1655 (41.0)		0.59 (0.33,1.04)
> 24 min	1466 (32.4)	148 (29.8)	1318 (32.7)		0.55 (0.31,0.98)

median (10th,90th percentile) or number (percent); * number missing

^a^ Unique patients, randomly selected occasions included

### Multivariable analysis of factors independently associated with 30-day mortality using multiple imputations (4587 patients, 500 died within 30 days)

The following were significantly independently associated with an increased risk of death: increasing age, a history of renal disease and cancer, an abnormal respiratory rate, a low oxygen saturation, a low systolic blood pressure, a decreased level of consciousness, and an abnormal body temperature. Only a history of pulmonary disease was associated with a decreased risk (Table 5).

**Table 5:** Multivariable analysis of factors independently associated with 30-day mortality using multiple imputations (4587 patients, 500 died within 30 days)

	Multiple imputations (500 + 4087)	
	OR^a^ (95%CI) ^a^	p
Age		< 0.0001
< =65	1	1
66–80	2.57 (1.76,3.75)	
> 80	3.68 (2.56,5.28)	
Pulmonary disease	0.68 (0.55,0.84)	0.0004
Renal disease	1.46 (1.11,1.91)	0.007
Cancer	1.81 (1.45,2.25)	< 0.0001
Respiratory rate < 8 or > 25 (breaths/min)	1.52 (1.21,1.90)	0.0003
Saturation < 90 (%)	2.61 (2.10,3.24)	< 0.0001
Systolic blood pressure < 90 (mmHg)	5.13 (3.18,8.27)	< 0.0001
Body temperature < 35.0 or > 41.0 (°C)	4.41 (1.78,10.96)	0.001
Degree of consciousness (RLS) > 2	3.61 (2.08,6.29)	< 0.0001

^a^ Odds ratio with corresponding confidence interval

## Discussion

Among all EMS-assigned patients that were classified as having dyspnoea as the main symptom by the PENs, 11.5% had a time-sensitive diagnosis, which means that the same patient could have had a time-sensitive diagnosis more than once. Among all EMS-assigned unique patients, approximately 13% had time-sensitive final diagnoses and among them approximately 27% died within 30 days.

The most important information was that aspects of the patients previous history and deviation from normal in several vital parameters were independently associated with both a time-sensitive final diagnosis as well the risk of death within 30 days. But symptoms of pain, a pathologic ECG, and a short delay from onset of symptoms until the call for EMS were significantly associated only with a time-sensitive diagnosis, whereas increasing age was associated only with the risk of death but not a time-sensitive diagnosis.

The finding that increasing age was significantly associated only with death but not with a time-sensitive diagnosis indicates that different mechanisms operate in attaining either of these endpoints. It may be that some of the time-sensitive diagnoses are particularly dangerous among the elderly. It may also be that some other diseases, which are not defined as time-sensitive diagnoses may still be life-threatening among the elderly. Previous studies [12, 13] emphasize that increasing age and especially older elderly (> 80 years) are more vulnerable, in addition to experiencing severe conditions. They are even more fragile due to their reduced capacity in managing their care and suffer from a decline in physiological reserves. Also, their physiological coping strategies in managing severe conditions may function differently, depending on how affected they are by their comorbidity.

A history of either hypertension or renal disease were significantly associated with a time-sensitive diagnosis. In the general population, the risk of hypertension increases with age [14, 15] and uncontrolled hypertension is a powerful and independent risk factor for cardiovascular morbidity and mortality as well as all-cause death. Hypertension is known as the *silent killer* because it has no specific symptoms and no early warning signs [14, 16–18].

The renal function decreases with increasing age and the prevalence of chronic kidney disease (CKD) increases steadily among people aged > 65 years [19–21].This is associated with an increased risk of death as well as cardiovascular disease and progression to renal failure [21, 22]. Sustained hypertension may lead to declines in kidney function and progressive failure in kidney function can conversely lead to worsening blood pressure control. As a consequence, it can lead to fluid accumulation inside the body with dyspnoea as a symptom that may persist despite optimal care in these patients [23–28]. Hypertension in combination with CKD poses a high risk for adverse outcomes depending on the severity level of renal failure [29–31].

A low systolic blood pressure predicted death within 30 days. Similar findings have been seen in EMS patients with chest pain, where a low systolic blood pressure was a strong predictor of acute- life-threatening conditions [32], as well as a predictor of death within 30 days [33]. A low systolic blood pressure is a hallmark of critical illness [34, 35] and a predictor of poor outcome in conditions such as acute myocardial infarction, sepsis or pulmonary embolism (where dyspnoea may be a presenting symptom) [34]. The assessment of the severity of pulmonary embolism is particularly based on the presence of hypotension or signs of shock and respiratory failure [36–38].

An abnormal body temperature (< 35 °C or > 41 °C) was significantly associated with death within 30 days. One reason may be physiological changes among the elderly, which tend to reduce the ability of organ systems to adapt to pathological changes. Thus, body temperature tends to be lower and the ability of the body to change with different stressors is reduced. Therefore, fever in an older patient often indicates a more severe infection and is associated with life-threatening consequences [15, 39].

A decreased level of consciousness was significantly associated with both death and a time-sensitive diagnosis. In unselected acutely and/or critically ill patients admitted to ED [35, 40], decreased level of consciousness was the most important predictor of early death. In EMS patients diagnosed with respiratory diseases in hospitals [6], the 30- day mortality was highest if decreased level of consciousness.

Reasons for decreased level of consciousness among patients with dyspnoea could be related to prolonged hypoxia and/or increased levels of carbon dioxide (CO_2_) [41, 42]. An abnormal respiratory rate is a more sensitive marker than other VS in identifying critically ill patients, as the body attempts to correct hypoxaemia and hypercarbia, by increasing respiratory rate. An abnormally low respiratory rate is often associated with a reduced level of consciousness [43].

A low oxygen saturation was associated with adverse outcomes in agreement with findings in medical patients in EDs [35, 44]. Previous results [35] claim that pulse oximetry lacks specificity and is therefore not a specific indicator for serious illness, since it does not mirror the quality of respiration and ventilation as it does not give information about the carbon dioxide (CO_2_) status.

Experiences from critically ill patients [40] indicate that the number of abnormal VS is associated with the risk of death. However, in patients with sepsis [45], VS may *only* be vague in prediction of the disease and can be normal. Instead, other symptoms and signs are common, (e.g. pain, acute altered mental status, leg weakness, and dyspnoea). In our study, only 39% had a pulmonary cause of the underlying infection, which indicated that dyspnoea is frequent in sepsis. This could indicate that the presence of dyspnoea is a part of a systemic pathophysiological response to the underlying infection, which may include an anaerobic metabolism and metabolic acidosis.

ECG pathology may be one of the most important signs predicting adverse outcome [32] and therefore a cornerstone in early detection of a time-sensitive diagnosis such as myocardial infarction [46]. ECG also have an important role in detecting severe arrhythmias, which can be the cause of dyspnoea and may lead to a time-sensitive diagnosis if not noticed [47]. A large proportion had a history of atrial fibrillation [8], which indicated that it was not a new finding that caused adverse illness among these patients.

The fact that pain was associated with a time-sensitive diagnosis is not unexpected, as this is previously described [38, 48, 49]. Pain may be due to the loss of perfusion (angina, myocardial infarction) and low oxygen tension (hypoxia) with resulting inflammatory response [50].

### Strengths and limitations

A strength is the large sample size, and the fact that all records were manually reviewed. The study is limited to southwest part of Sweden, which could hamper generalisability to other settings. Thus, in the north of Sweden there are longer transport distances which may lead to fluctuations. Furthermore, data were retrospectively collected from patient records and VS and other clinical parameters could have been measured but never reported. There was sometimes insufficient documentation. Dyspnoea could be present in other medical conditions such as chest pain. Thus, some patients with dyspnoea may have been classified to other ESS codes (not ESS 04).

The final diagnosis was missing in 1030 cases explained by patients left on-scene and patients transported to the ED who were directly sent home. However, with the intention to reduce bias associated with medical record reviews, the study was conducted in agreement with the paper by Kaji et al. [51].

## Conclusion

Among patients with dyspnoea as the main symptom, several factors including age, previous medical history, deviating VS, the ECG pattern, symptoms of pain and the delay between onset of symptoms and the call for EMS are important to consider in the early risk assessment. The development of a decision support tool may increase the possibility to differentiate patients with time-sensitive conditions, from those without, at an early stage.

## Supplementary Information

**Additional file 1.** Deviating vital signs Red/Orange level according to RETTS-A (2017 version).

## Data Availability

The datasets analysed during the current study are available from the corresponding author on reasonable request.

## Abbreviations

CKD: Chronic kidney disease
ECG: Electrocardiogram
ED: Emergency department
EMS: Emergency medical services
ESS: Emergency Symptoms and Signs
ICD-10: International Statistical Classification of Diseases and Related Health Problems – 10th revision
PEN: Pre-hospital emergency nurse
RETTS-A: Rapid Emergency Triage and Treatment System for adults
VS: Vital signs
